# A Longitudinal Emergency Medical Services Track in Emergency Medicine Residency

**DOI:** 10.7759/cureus.1127

**Published:** 2017-03-30

**Authors:** Daniel Adams, Jason Bischof, Ashley Larrimore, William Krebs, Andrew King

**Affiliations:** 1 Emergency Medicine, The Ohio State University Wexner Medical Center

**Keywords:** emergency medicine services, emergency medicine, education, curriculum development

## Abstract

Emergency medicine residency programs offer Emergency Medical Services (EMS) curricula to address Accreditation Council for Graduate Medical Education (ACGME) milestones. While some programs offer advanced clinical tracks in EMS, no standard curriculum exists. We sought to establish a well-defined EMS curriculum to allow interested residents to develop advanced clinical skills and scholarship within this subspecialty.

Core EMS fellowship trained faculty were recruited to help develop the curriculum. Building on ACGME graduation requirements and milestones, important elements of EMS fellowship training were incorporated into the curriculum to develop the final document.

The final curriculum focuses on scholarly activities relating to the four core areas of EMS identified by The American Board of Emergency Medicine and serves as an intermediary between ACGME graduation requirements for education in EMS and fellowship level training.

Standardization of the EMS scholarly track can provide residents with the potential to obtain competency beyond ACGME requirements and prepare them for success in fellowship training and/or leadership within EMS on graduation.

## Introduction

Emergency Medical Services (EMS) provides the initial medical evaluation and treatment for patients with acute medical emergencies. Patients with emergent conditions benefit from initial triage, treatment, and stabilization performed by pre-hospital providers resulting in both improved short and long-term clinical outcomes [1-2]. Traditionally, EMS medical oversight has been provided by community physicians, including emergency medicine physicians. As the scope and utilization of EMS services continue to grow, there is an increased need for physicians with an understanding of the unique pre-hospital clinical practice environment. Accordingly, the American Board of Medical Specialties recognized EMS as a subspecialty of emergency medicine in 2010. The subspecialty of EMS standardizes physician training and qualifications for the practice of pre-hospital medicine. It is intended to enhance the quality of care provided to patients in the pre-hospital setting and to facilitate the integration of pre-hospital treatment into the continuum of care [3]. As the clinical interplay between the hospital and pre-hospital providers continues to strengthen, it is increasingly important to train emergency medicine residents to assume leadership roles in the management of EMS. Preferably, this would include focused education in the subspecialty of EMS to include fellowship training.

Emergency medicine training programs need a defined foundational curriculum in EMS, including on and off-line medical control, medico-legal aspects, communications, disaster management, and EMS structure, to ensure graduation of physicians competent in EMS medicine. Despite prior publications relating to EMS curricular design and the minimum training requirements determined by the Accreditation Council for Graduate Medical Education (ACGME) for emergency medicine residents specific for EMS, the training experiences completed by residents are variable [4-5]. Such variability leaves open the opportunity to provide our residents with a robust and well-defined curriculum that may lead to better preparation upon graduation or interest in EMS as a fellowship.

As with the specialty of emergency medicine, it will be years before the supply of EMS subspecialists meets the demand. Rural areas and uncompensated or poorly compensated EMS coverage areas will continue to face challenges in the recruitment of EMS trained medical directors. The National Association of EMS Physicians (NAEMSP) position statement on physician medical direction in EMS recommends that physicians be board certified or board prepared in emergency medicine with an active clinical practice and completion of an EMS fellowship. In addition, the NAEMSP statement lists training or significant experience in the clinical practice of pre-hospital EMS and the provision of both direct and indirect medical direction as requirements for EMS medical directors. The statement includes the requirement of knowledge in the broad array of pre-hospital medicine design, laws, and operation that exceed the minimum training required by the ACGME for emergency medicine residency completion [6]. Overall, the vast majority of graduating residents will not have the necessary skills to effectively lead an EMS agency, and we should seek to spark interest and foster leadership early on within residency training.

Resident education and interest are the key to improving this situation. Presently, many ACGME graduating residents fail to meet NAEMSP recommendations for EMS directorship. Therefore, we need to enhance resident education and interest in this subspecialty. By providing interested emergency medicine residents a focused track in EMS during their training, we might see improvement in not only the number of graduates pursuing fellowship training but also success and productivity of EMS fellows. For residents who choose not to pursue fellowship training, niche based training during residency may strengthen their job application and foster interest in local involvement in EMS medicine [7].

The purpose of the curriculum proposed in this report is to describe a structured curriculum that bridges the gap between minimum ACGME requirements for emergency medicine residents in EMS and the requirements for EMS fellowship training. The goal of our longitudinal scholarly track in EMS medicine is to promote scholarship within EMS while encouraging residents to pursue continued training within the subspecialty. 

## Technical report

Core education faculty members involved in our EMS curriculum were recruited along with interested residents to structure a scholarly track based on our current experience, ACGME requirements, and NAEMSP guidelines. A local needs assessment is institutionally variable in regards to available and necessary resources. At our institution, we identified EMS fellowship trained faculty mentorship, developed dedicated and available didactic time, and identified local EMS agencies able to participate in the curriculum. To ensure the curriculum was goal-directed, clear goals (Table 1) and objectives with ACGME milestones addressed and educational methods (Table 2) were incorporated into the structure of the track.

The final curriculum was established with additional requirements in the four core areas of EMS medicine: Clinical Aspects of EMS Medicine, Medical Oversight of EMS, Quality Management and Research, and Special Operations. The curriculum includes administrative, research, clinical, and educational domains expanding upon the basic residency requirements outlined by the ACGME (Table 2).

Collectively, the advanced track curriculum includes an additional 36 hours of EMS didactics, completion of defined EMS-related competencies, and required scholarly and quality improvement projects focused on EMS. Didactic sessions were based upon the Ohio American College of Emergency Physicians Medical Director's Course and the selected textbook readings provided in the appendix.

Learners will receive biannual formative evaluation letters completed by track mentors providing detailed feedback in regards to track participation and specific opportunities for improvement. An annual summative review evaluation letter will be provided highlighting their performance and completion of scholarly track requirements. Ongoing annual assessment of the program will be performed by a focus group consisting of resident participants, residency program leadership, and track mentors. Identified opportunities for improvement will be addressed by a specific detailed plan developed by the focus group.

**Table 1:** Goals of the EMS Scholarly Track EMS: Emergency medical services.

Goals
Develop the skills and knowledge required to become an EMS service medical director following successful completion of the EMS scholarly track
Acquire the knowledge and skills to begin an EMS fellowship following completion of the track
Contribute to the field of EMS through research, scholarly production, education, and policy development
Demonstrate the ability to pursue personal interests in EMS, including disaster medicine, search and rescue, hazardous materials, aeromedicine, and tactical medicine

**Table 2:** EMS Scholarly Track Objectives and Associated Milestones *Teaching methods: small group discussion, lecture, EMS medical direction, simulation EMS: Emergency medical services; ICS: Interpersonal communication skills; MK: Medical knowledge; PROF: Professionalism.

Clinical Aspects of EMS Medicine*	Medical Oversight of EMS*	Quality Management (QI) and Research*	Special Operations*
Actively participate in the provision of care in the pre-hospital setting (MK, PC, PROF, ICS)	Provide attending supervised medical direction for all requests for online medical control by pre-hospital care providers (MK, PC, PROF, ICS)	Participate in the QI system of the assigned agency, including attendance at regular QI meetings, as well as addressing specific issues as identified by the agency’s medical director, QI supervisor, pre-hospital care personnel, or the resident (MK, PC, PROF, ICS)	Participate and help run disaster/mass casualty/mass gathering exercises with their assigned agency as well as the various health systems
Explain resource allocation, dispatch issues, pre-arrival instructions (MK, PC, PROF, ICS)	Assist the continuing education of EMS personnel (MK, PC, PROF, ICS)	Perform one QI project directly related to the agency’s interface with the Emergency Department; this project will fulfill the residency QI project requirement (MK, PC, PROF, ICS)	
Demonstrate proficiency in pre-hospital specific procedures not routinely covered as a part of residency training (MK, PC, PROF, ICS)	Assist the Center for EMS Education including skills sessions, didactic lectures, drills, simulation/scenario, and examinations (MK, PC, PROF, ICS)	Perform one research project of sufficient complexity and depth agreed upon between the EMS faculty and resident; this project will fulfill the residency scholarly project requirement (MK, PC, PROF, ICS)	
Assist the assigned agency with company training in-services on new equipment, protocols, and policies as requested by the agency (MK, PC, PROF, ICS)	Assist EMS faculty in developing and delivering core EMS curriculum for non-EMS track residents (MK, PC, PROF, ICS)		

## Discussion

The ACGME requirements for EMS training during an emergency medicine residency help establish a minimum competency in this domain. The implementation of an EMS specific advanced track curriculum can augment a program’s traditional curriculum and provide expanded opportunities for resident learners [8]. Educational longitudinal tracks within the emergency medicine curriculum have been established in a multitude of emergency medicine subspecialties. Regan, et al. describe the benefits of offering scholarly tracks within the emergency medicine residency curriculum to include increased competitiveness of applicants for fellowship and academic faculty positions, development of expertise in a clinical niche, improved focus and purpose of departmental scholarly output, increased resident interest in an academic career, increased faculty development through mentorship of residents participants, and increased volume and quality of scholarly output [8]. An EMS scholarly track will hopefully lead to more residents completing EMS fellowship and leadership within this domain. At a minimum, an EMS advanced track should lead to graduates capable of contributing to the oversight and training of pre-hospital providers and participating in the overall direction of local community EMS medicine capabilities. It should be noted, however, that the track is not meant to replace EMS fellowship training.

The proposed curriculum presented builds upon ACGME emergency medicine requirements and mirrors aspects of the EMS Fellowship Guidelines [9]. The EMS scholarly track bridges the standard emergency medicine residency curriculum to a topic-focused training experience received during a fellowship program. We chose to divide the requirements into categories, which focuses learners and educators to facilitate expectations. Focusing on these broad categories engages residents in quality improvement efforts, curriculum preparation and revisions, delivery of educational content to colleagues and pre-hospital providers, and scholarly projects that prepare residents for the fellowship experience.

We believe that in using the aforementioned format, we will produce residents with an enhanced competence in EMS, either ready to enter the workforce as a leader and participate in the activities of local EMS agencies or to excel in EMS fellowship programs. There are some emergency medicine residency programs that have EMS scholarly tracks in place, but no published standard curriculum exists. As with any curriculum, ongoing evaluation and refinement will be required [10]. Future work on our proposed curriculum will require additional data to validate the proposed benefits of track implementation in regards to resident experience, resident performance, and EMS-based departmental scholarly productivity.

### Limitations

The main limitation of our curriculum design is the lack of prospective data looking specifically at outcomes such as resident satisfaction, scholarly output, or numbers of residents who graduate seeking fellowship in EMS or leadership opportunities in the field. Because the needs assessment is specific to our program, it will be difficult to generalize to other institutions; therefore, each program should perform its own needs assessment based on available resources. Moving forward, it is our goal to look at such data. We also hope to collaborate with other institutions to ultimately develop a best practice for broad application.

## Conclusions

A focused EMS scholarly track permits interested residents to acquire further experience in EMS medicine, including the administrative tasks required in practice. Although a subset of residents may continue to build on the knowledge base acquired through an EMS advanced track via a dedicated EMS fellowship, a scholarly track allows others to graduate residency with an enhanced ability to further develop their professional goals and serve as leaders in their local EMS medicine community. Given that no standard EMS advanced track curriculum exists, we attempted to define such a curriculum for standardized implementation. Future studies assessing the impact of the curriculum on learner satisfaction and EMS-based competency will be performed to determine areas of curricular improvement.

## Appendices

### Education materials

Ohio ACEP EMS Medical Directors Course^1^

Emergency Medical Services: Clinical Practice and Systems Oversight – Second Edition

     Volume 1

          1. History of Emergency Medical Services 2. Airway Management 3. Airway Procedures 4. Airway Management: Special Procedures 11. Cardiac Arrest Systems of Care 26. Trauma Systems of Care 39. Field Trauma Triage 53. The Special Needs of Children 54. Pediatric Medical Priorities 55. Pediatric Trauma Priorities 56. Technology Dependent Children 57. Approach to the Geriatric Trauma Patient 58. Bariatric Patient Challenges 63. Ethical Challenges 64. End of Life Issues 65. Termination of Resuscitation in the Out of Hospital Setting 66. Family and Bystanders 70. Culture of Patient Safety 72. Defining, Measuring, and Improving Quality 73. Data Management and Information Systems 74. EMS Quality Improvement and the Law

     Volume 2

          1. Principles of EMS System Design 2. Air Medical Services 3. Interfacility Transportation 4. Legislation, Regulation, and Ordinance 6. EMS Personnel 8. Medical Oversight of EMS Systems 10. EMS Dispatch 12. Emergency Care Regionalization 16. Legal Issues 18. Risk Management 19. EMS Provider Education 26. Incident Command System and National Incident Management System 27. Medical Management of Mass Gatherings 44. EMS Research Basics 45. Informed Consent for EMS Research 46. Cardiac Arrest Related Research Methodology 47. Trauma Related Research Methodology 48. Pediatric Related Research Methodology 49. Economic Evaluation of EMS Related Interventions 50. Handling and Statistics Essentials

^1^http://ohacep.org/aws/OACEP/asset_manager/get_file/70188/ems_md_course_2013.pdf?ver=19495
